# MiR-145 reduces ADAM17 expression and inhibits *in vitro* migration and invasion of glioma cells

**DOI:** 10.3892/or.2012.2084

**Published:** 2012-10-17

**Authors:** YONG LU, MICHAEL CHOPP, XUGUANG ZHENG, MARK KATAKOWSKI, BENJAMIN BULLER, FENG JIANG

**Affiliations:** 1 Department of Neurology, Henry Ford Hospital, Detroit, MI; 2 Department of Physics, Oakland University, Rochester, MI, USA

**Keywords:** miR-145, ADAM17, EGFR, glioma, migration, invasion

## Abstract

MicroRNAs are important regulators of gene expression and have been suggested to play a key role in tumorigenesis. In this study, we show that miR-145 is significantly downregulated in glioma cell lines compared to normal brain tissue and negatively regulates tumorigenesis. Restoration of miR-145 in glioma cells significantly reduced *in vitro* proliferation, migration and invasion. Also, overexpression of miR-145 reduced ADAM17 and EGFR expression. In addition, we tested the hypothesis that the miR-145-mediated suppression of cell proliferation, migration and invasion is, at least in part, due to silencing of ADAM17 and EGFR gene expression. Using luciferase reporters carrying the 3′-untranslated region of ADAM17 combined with western blotting, we identified ADAM17 as a direct target of miR-145. Collectively, these results suggest that as a tumor suppressor, miR-145 inhibits not only tumor proliferation, but also cell migration and invasion, and warrants further investigation.

## Introduction

MicroRNAs (miRNAs) are short single-stranded nucleotide RNA molecules, which function as master regulators of gene expression by post-transcriptional modifications of target mRNAs (1). The pattern of regulation of gene expression is sequence-specific. MiRNAs bind to 3′ untranslated regions (3′-UTRs) of mRNAs and then reduce the translation and/or stability of that mRNA, leading to a reduction in protein levels. Based on the unique feature of their targeting, miRNAs could have many targets (2), and, thus, control a large number of proteins.

MiRNAs may serve as either oncogenes or tumor suppressors (3,4). In our study, the miR-145 is a tumor-suppressive miRNA (5) and its expression is low in different kinds of cancers. Mir-145 inhibits proliferation by targeting c-myc (6) and MUC1 (7). In addition, miR-145 can also promote differentiation and repress cell growth by repressing OCT4 (8).

ADAMs are best known as ectodomain sheddases, and their domains function as metalloproteases. The ADAM family is a Zn-dependent metalloproteinase (9,10). ADAM17 is an important member of the ADAM family and involved in proteolysis of collagen IV of the extracellular matrix and also the release from the cell surface of several integrins, suggesting that ADAM17 influences the invasive activity of different cells including glioma cells (11). ADAM17 is a primary upstream component for multiple EGFR pro-ligands (12,13). EGFR binding with ligands subsequently activates MEK/ERK and PI3K/Akt pathways, which contribute to invasiveness and other malignant phenotypes (14).

In this study, the expression of miR-145 in glioma cells compared with normal brain tissue was studied by real-time RT-PCR. Proliferation, migration and invasion were examined to confirm the effects of miR-145 in glioma cells. The suppression of ADAM17 by miR-145 was confirmed by experiments using luciferase analysis and western blotting. We found that ectopic expression of miR-145 in U87 and U251n glioma cells caused decreased proliferation, migration and invasion with accompanying low protein expression of ADAM17 and EGFR. Our study provides direct evidence that miR-145 functions as an anti-oncogene in glioma cells and may be the target of therapies for glioma patients.

## Materials and methods

### Cells and miRNA transfection

U87-MG, U251n and T98G cells were obtained from the American Type Culture Collection (Manassas, VA, USA). HF66 cells were established by the Neurosurgical Department of Henry Ford Hospital. Cells and were maintained in Dulbecco’s modified Eagle’s medium (DMEM) supplemented with 10% fetal bovine serum (FBS). Total RNA from human frontal cortex brain tissue were obtained from Agilent Technologies (Santa Clara, CA, USA). Transfection of the miRIDIAN hsa-miR-145 miRNA mimic (Applied Biosystems), inactive (scrambled) control cel-mir-67 (Thermo Scientific Dharmacon, IL, USA), or pMIR-Report vectors was performed using Lipofectamine 2000 transfection reagent (Invitrogen, CA, USA) with 300 nmol of miRNA or 1 μg/ml DNA plasmid, respectively.

### Western blot analysis and real-time RT-PCR

Western blot analysis was performed to detect ADAM17 (Abcam, MA, USA), EGFR, p-EGFR (Santa Cruz Biotechnology, Santa Cruz, CA, USA), and β-actin (Santa Cruz). Protein expression was measured 72 h after transfection. Protein concentration was quantified using a BCA protein assay kit (Pierce, Rockford, IL, USA). For quantitative analysis, densitometric measurements of three blots per group were averaged. Densitometry was performed with the MCID image analysis program (Cambridge, UK). The image density (1/intensity) of each band was normalized to β-actin, and experimental group band density was normalized to the band density of control. RT-PCR for miRNAs was performed using a miR-145 TaqMan MicroRNA assay. U6 miRNA was used as a house-keeping control. Each sample was tested in triplicate and relative gene expression was determined.

### Invasion assay

Matrigel chambers (BD Biosciences) were used to determine the effect of miR-145 on invasiveness according to the manufacturer’s instructions. Cells (5×10^4^), after being transfected with miR-145 for 72 h, were re-suspended in 500 μl of serum-free medium and added to the upper chamber while the lower chamber was filled with 0.5 ml of complete medium that served as a chemo-attractant. Cells were then incubated for 24 h at 37°C. After removal of cells on the upper surface of the membrane, cells on the lower surface of the membrane were stained with CellTracker™ Green (Molecular Probes, Eugene, OR, USA) for 45 min and fixed in 4% formaldehyde. Nine fields of cells were counted randomly in each well under a fluorescent microscope at magnification ×100. All the experiments were done in duplicate and results were expressed as mean ± SEM of three independent experiments.

### Migration assay

After transfection, cells were seeded in 12-well plates in complete medium. When the cell confluence reached ~90%, at 72 h post-transfection, an artificial circle wound was made onto the monolayer with a 1-ml micropipette tip. The wound area was then examined after 24 h of incubation under an optical microscope at magnification ×100. Photographs were taken and the cell migration ability was expressed by the gap closure.

### Proliferation assay

Cell proliferation was measured using an ELISA kit (Roche Applied Science, Germany) according to the manufacturer’s instructions. Briefly, 4×10^3^ cells were plated to each well of a 96-well plate after transfection, and then cells were incubated with bromodeoxyuridine for 2 h. The cells were subsequently fixed and incubated with 100 μl of detection antibody conjugated with peroxidase and substrate, respectively. Light emission of the samples was measured in a microplate luminometer.

### MirRNA luciferase assay

Position 416–422 of the ADAM17 3′-UTR is a predicted interaction position of miR-145. We cloned an 80-bp sequence containing the predicted binding site or a scrambled sequence downstream of the pMIR luciferase reporter to generate pMIR-ADAM17 and pMIR-mut-ADAM17 vectors, respectively. Luciferase assays were carried out in U87 cells. First, cells were co-transfected with appropriate plasmids with either hsa-miR-145 miRNA mimic or control cel-mir-67 mimic in 12-well plates. Then, the cells were harvested and lysed for luciferase assay 24 h after transfection. Luciferase assays were performed using a luciferase assay kit (Promega) according to the manufacturer’s instructions.

### Statistical analysis

Data are presented as mean and standard error (mean ± SEM). Statistical significance was analyzed by one-way ANOVA using the GraphPad Prism software (version 4.0). P-value smaller than 0.05 (P<0.05) was considered significant.

## Results

### Expression profile of miR-145 in glioma cells

**Figure 1:**
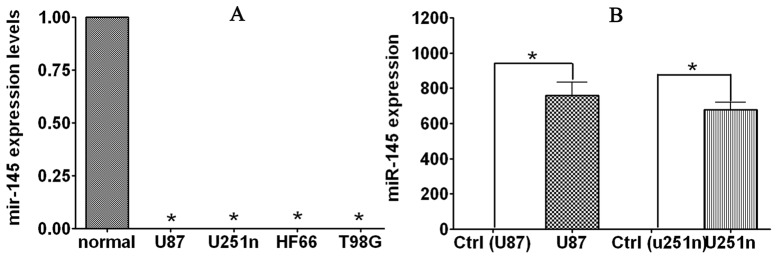
mir145 expression levels in glioma cell lines. (A) Total RNA was isolated from the normal brain tissue and glioma cells of U87, U251n, HF66, and T98G and real-time PCR was employed to analyze the expression of miR-145. The relative expression of miR-145 was expressed as the ratio of the expression level of normal brain tissue. ^*^P<0.01, as compared with normal brain tissues. (B) Glioma cells were transfected with miR-145 mimic for 72 h, and then collected for real-time RT-PCR. The expression of miR-145 was significantly increased in U87 and U251n, as compared with non-transfected cells. ^*^P<0.01, as compared to glioma cells without transfection of miR-145.

A growing body of studies report that miR-145 is downregulated in cancers (5,15–19). However, the expression of miR-145 in glioma has not been well documented (20,21). We sought to identify the role of miR-145 in human glioma cells. We compared the expression levels of miR-145 in glioma cell lines with total RNA from frontal cortex using real-time RT-PCR. As shown in the Fig. 1A, miR-145 expression levels were significantly decreased in tumor cell lines compared to total RNA from normal brain tissues. Expression of miR-145 in U87, U251n, T98G and HF66 glioma cell lines 0.113, 0.002, 0.045 and 0.002% of normal brain tissue. The reduced expression of miR-145 in glioma cells suggests that miR-145 is a potential target in glioma therapy.

### MiR-145 overexpression inhibits proliferation of glioma cells

**Figure 2:**
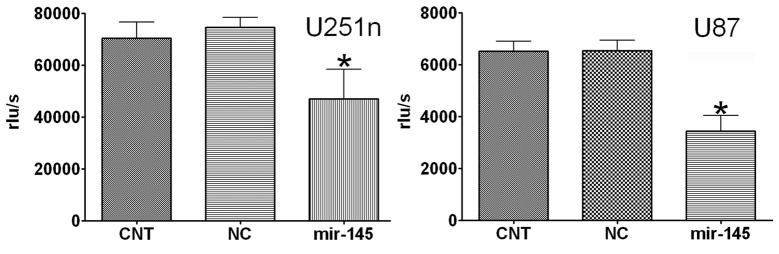
Overexpression of miR-145 reduces glioam cell proliferation *in vitro*. Glioma cells were transfected with miR-145 and negative control. After incubation for 72 h, cell proliferation rates were analyzed by BrdU assay. ^*^P<0.01, as compared to control and negative control.

To test the effect of miR-145 on cell growth, we used miR-145 precursor microRNA to infect human glioma U251n and U87 cells. After transfection, miR-145 levels were increased in both cell lines, indicating that enhancement was due to the introduction of precursor miR-145 (Fig. 1B). As demonstrated by BrdU assay, overexpression of miR-145 significantly reduced cell proliferation by >40% in U87 and U251n as compared to cells transfected with control (Fig. 2). These data suggest miR-145 has anti-proliferative effects on U87 and U251n *in vitro*.

### Ectopic expression of miR-145 inhibits glioma cell migration and invasion

**Figure 3:**
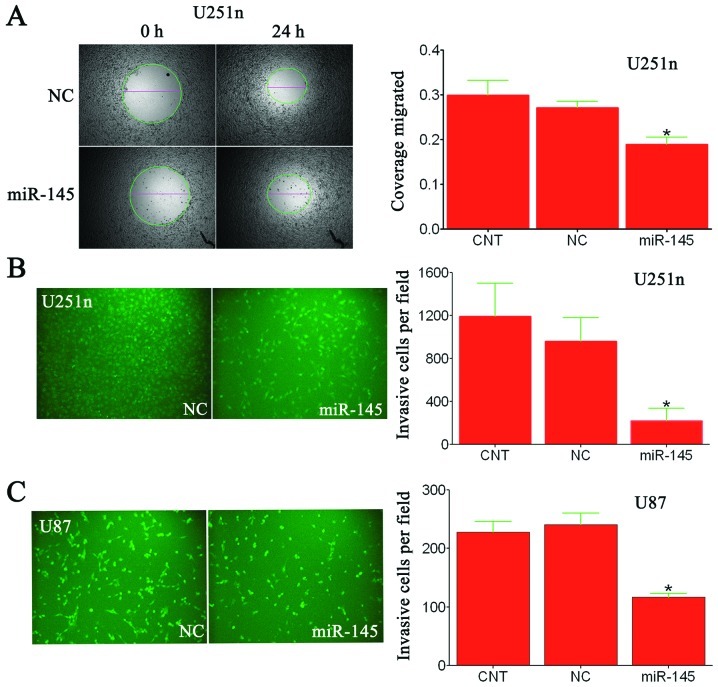
Effect of miR-145 overexpression on cell migration and invasion in U251n and U87. (A) Wound healing assay was performed to evaluate the effects of miR-145 on glioma cell migration. U251n cells were transfected with miR-145 at a final concentration of 300 nM. When the cell confluence reached ~90% at 72 h post-transfection, an artificial homogeneous wound was made as described in Materials and methods. The experiments were preformed in triplicate and the representative images of photographs at 0 and 24 h post-wounding are shown at magnification ×100. ^*^P<0.01, as compared to control and negative control. (B) Effect of miR-145 overexpression on glioma cell invasion. Glioma cells transfected with miR-145 or negative control were plated on Matrigel-coated membranes in the upper chamber of transwells. After incubation for 24 h, non-invading cells on the upper surface of the membrane were removed and the invasive cells on the lower surface were stained with cell tracker. The stained invasive cells were photographed under a fluorescence microscope (magnification ×200). ^*^P<0.01, as compared to control and negative control. (C) To confirm the effect of miR-145 overexpression on glioma cells invasion, we employed the cell line U87. U87 cells were also transfected with miR-145 or negative control, and then analyzed with Matrigel-coated membranes chamber invasion assay. ^*^P<0.01, as compared to control and negative control.

Previous studies reported that miR-145 decreases invasion and migration of breast cancer cells *in vitro* and *in vivo*(7). ADAM17 and EGFR have been reported to play important roles in glioma, and overexpression of ADAM17 promotes glioma invasiveness (22,23). To determine if miR-145 had a similar effect on glioma cells, we employed a wound healing migration assay and a Matrigel invasion chamber assay. Seventy-two hours after transfection with miR-145 or scrambled-miRNA, U87 and U251n cells were seeded into the upper chamber, and then cells that invaded through the extracellular matrix after 24 h were imaged and counted (Fig. 3B and C). In both cell lines, miR-145 significantly decreased the number of cells that invaded compared to controls. Similarly, using a wound healing assay, we examined the effect of miR-145 on U251n cell migration (Fig. 3A). Here, we found miR-145 inhibited migration of U251n glioma cell migration, as compared with scrambled-miRNA transfected control groups. Taken together, these data indicate that miR-145 serves as a regulatory molecule involved in cell migration and invasion *in vitro*.

### ADAM17 and EGFR as targets of miR-145 for post-transcriptional repression

**Figure 4:**
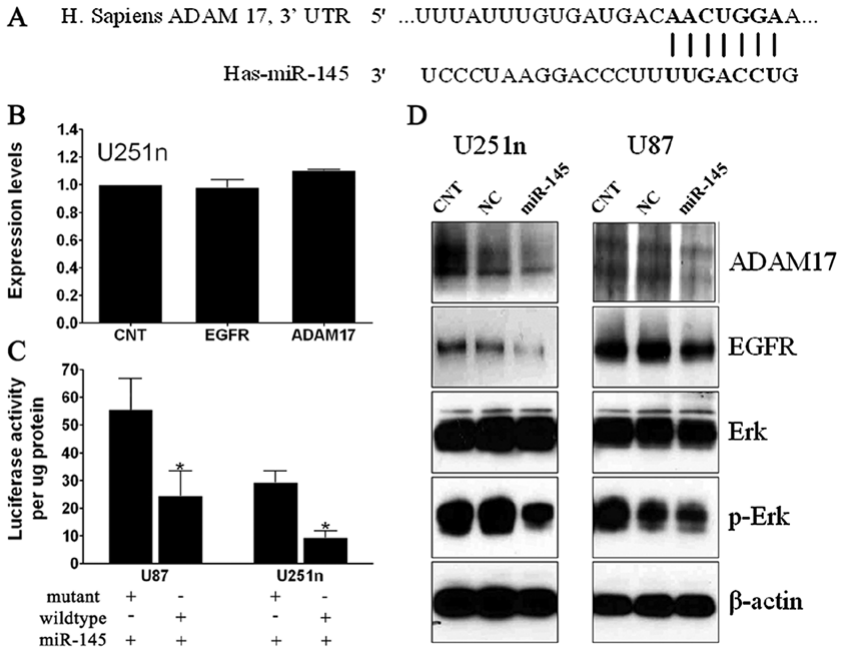
ADAM17 is a predicted target of miR-145 for post-transcriptional repression. (A) The miR-145 seed sequences and their predicted binding sites in the ADAM17 3′-UTR are shown. The highlighted region representing the binding site was predicted by Targetscan. (B) The mRNA expression of ADAM17 and EGFR in glioma cells. Overexpression of miR-145 did not decrease the mRNA expression of EGFR and ADAM17. (C) MiR-145 targets the predicted binding site within the ADAM17 mRNA 3′-UTR, decreasing luciferase in U87 and U251n cells transfected with a luciferase reporter vector containing the predicted binding site. Mutant-ADAM17, vector containing mutated binding site; wild-type-ADAM17, vector containing predicted binding site. ^*^P<0.01 compared to U87 or U251n transfected with vector containing a mutated ADAM17 site and miR-145. (D) The protein expression of ADAM17 and EGFR in U251n cells. Western blot analysis was employed to test the expression of ADAM17 and EGFR in glioma cells. β-actin was used as an internal standard.

Given that miR-145 reduces migration and invasion of U251n and U87 glioma cells, we then sought to identify the target genes of miR-145 using the online miRNA target prediction programs Targetscan and Pictar (Fig. 4A). Approximately 150 targets of miR-145 were predicted from these programs. ADAM17 was of particular interest because our previous studies revealed that its expression level was upregulated in glioma specimens. ADAM17 overexpression contributes to glioma progression (23). It was recently reported that miR-145 inhibits expression of EGFR and reduces cell growth of lung cancer cells (24).

To test whether miR-145 suppresses the expression of ADAM17 and EGFR at protein level in glioma, we performed Western blot analysis. Employing a ‘scrambled’ cel-mir-67 or miR-145 mimic, we found that ADAM17 and EGFR protein expression were significantly reduced by miR-145 compared to miRNA controls in both cell lines (Fig. 4D). However, overexpression of miR-145 did not change mRNA expression of ADAM17 and EGFR (Fig. 4B). These data suggest that miR-145 targets ADAM17 and EGFR by post-transcriptional repression.

ADAM17 is a primary sheddase for multiple EGFR pro-ligands (25,26). EGFR is an important mediator responsible for the invasiveness of malignant gliomas (14,27). EGFR ligand binding results in receptor self-dimerization, autophosphorylation and subsequent activation of downstream Ras/MAPK/ERK signaling pathways, which contribute to the malignant phenotype (28). As reduced protein expression of ADAM17 and EGFR by miR-145, Western blot analysis was also employed to determine the expression of downstream signaling protein Erk/p-Erk. Compared to controls, miR-145 has no effect on Erk, but significantly decreased p-Erk expression. These data indicate that miR-145 may reduce migration and invasion through the ADAM17/EGFR/Erk/p-Erk signaling pathway.

To further confirm that the effects observed above are ascribed to the specific interaction between miR-145 and the binding sites for miR-145 in the 3′-UTR of ADAM17, we performed a Luciferase activity assay. As shown in Fig. 4C, miR-145 suppressed >40 and 50% activity, respectively, of Luc-ADAM17-UTR (wild-type) in U87 and U251n compared with mutant ADAM17 3′-UTR, which suggest that miR-145 directly targets the 3′-UTR of ADAM17 mRNA.

## Discussion

In the present study we detected the miR-145 expression level in U87, U251n, T98G and HF66 human glioma cell lines, and found a significant reduction of miR-145 in human glioma cell lines compared to total RNA of frontal cortex. The expression level of miR-145 negatively regulates glioma malignancy. Ectopic expression of miR-145 decreased proliferation, migration, and invasion of glioma cells. These data suggest that miR-145 plays a critical role in glioma development, and it may function as an anti-tumor factor in glioma cells.

Our studies indicate that ADAM17 is a target gene of miR-145, and several lines of evidence support the direct interaction between miR-145 and ADAM17: i) the 3′-UTR of ADAM17 contains a binding site for miR-145 with significant seed match; ii) miR-145 suppresses the activity of a luciferase reporter with the 3′-UTR of ADAM17 mRNA; iii) miR-145 represses ADAM17 expression at protein level; (4) our previous studies have shown that ADAM17 knockdown by siRNAs resulted in a decreased glioma cell proliferation, migration and invasiveness. We therefore, the first time, identify miR-145 as directly regulating ADAM17.

ADAM17 expression is elevated in different cancers and is associated with cancer progression (29,30). High levels of ADAM17 have been reported in a variety of cell lines and clinical tissue samples, including breast, colon, prostate and glioblastoma (23,31–33). Our previous study showed that high level expression of ADAM17 was found in high grade glioma samples. Knockdown of ADAM17 inhibits breast cancer and glioma cell proliferation and invasion (23,31). Ectopic expression of ADAM17 induced tumorigenicity of cortical astrocyte cell line (34) and promotes both breast and glioma cell malignant phenotypes (23,31). In our present study, we demonstrated that miR-145 is an important regulator of ADAM17, and directly binds the 3′-UTR of ADAM17 mRNA in glioma cells. Thus, the reduction of miR-145 in glioma cells yields an increased expression of ADAM17.

One of the characteristics of glioma is invasiveness. The EGFR is over-expressed in ~50–60% of gliomas (35), and EGFR increases with malignancy grade, and is required for maintenance of glioma growth (36). EGFR serves as an aggressive agonist of glioma invasion (37). In our study, transfection of glioma cells with miR-145 resulted in decreased protein expression of EGFR, which may be partly responsible for the miR-145 induced decrease of glioma cell invasion, and consistent with a recent report that miR-145 decreased EGFR expression and proliferation in lung cancers (38). These data suggest that miR-145 may also target the EGFR gene. ADAM17 is involved in the ectodomain shedding of multiple membrane-bound ligands and cytokines, implicated in diverse biological processes, including growth and inflammation (23,39). Interestingly, ADAM17 has recently been identified as the primary sheddase for multiple EGFR pro-ligands. EGFR ligand-binding results in receptor self-dimerization, autophosphorylation and subsequent activation of downstream Ras/MAPK/ERK signaling pathways (28,40). Taken together with reduced p-Erk expression, miR-145 transfection may decrease glioma cell migration and invasion through ADAM17/EGFR/ERK signaling pathway.

In conclusion, our data suggested that miR-145 plays a key role in the malignancy of glioma cells possibly by direct regulation of ADAM17 protein expression, which affects glioma cell proliferation, migration and invasion. However, further study is needed to determine if ADAM17 activity is influenced by miR-145 *in vivo* in glioma.
